# Supplementary material for: Functional Characterization of Paillotin: An Immune Peptide Regulated by the Imd Pathway with Pathogen-Specific Roles in Drosophila Immunity
Source: Proc Biol Sci. Author manuscript; Available in PMC 2025 Nov 4. (PMC12419901; doi:10.1098/rspb.2025.1835)
Supplement: Supplementary Materials [file EMS209708-supplement-Supplementary_Materials.pdf]

bioRxiv preprint doi: https://doi.org/10.1101/2025.05.12.653313; this version posted July 25, 2025. The copyright holder for this preprint (which was not certified by peer review) is the author/funder, who has granted bioRxiv a license to display the preprint in perpetuity. It is made available under aCC-BY 4.0 International license.

Supplementary material for

Functional Characterization of Paillotin: An Immune Peptide Regulated by the Imd Pathway with Pathogen-Specific Roles in *Drosophila* Immunity

Yao Tian[1], Xiaojing Yue[3], Renjie Jiao[4], Mark A. Hanson[1,2]*, Bruno Lemaitre[1]*

1: Global Health Institute, Ecole Polytechnique Fédérale de Lausanne (EPFL), Lausanne, Switzerland

2: Centre for Ecology and Conservation, University of Exeter, Penryn, United Kingdom

3: Laboratory of Biology and Modelling of the Cell, Ecole Normale Superieure of Lyon, CNRS, Lyon, France.

4: Sino-French Hoffmann Institute, Guangzhou Medical University, Guangzhou, China

*: Equal last author: M.A. Hanson (M.Hanson@exeter.ac.uk), B.L. Lemaitre (bruno.lemaitre@epfl.ch)
DOI: 10.1098/rspb.2025.1835

Table S1: Collectively, 39 of 789 genome-sequenced fly strains, including 18 of the 205 *Drosophila* Genetic Reference Panel lines encode a loss-of-function mutation in *Paillotin*. Lines encoding *Pai* putative loss-of-function mutations are annotated, alongside the genetic description of each allele. $Pai^{\Delta 41}$ "uncertain" and "alternate" alleles per the DGRP variant call dataset are both represented by a string of ~61 uncertain nucleotide calls (Ns) matching the pattern of allele 2R_19488505_DEL in the DPGP3 dataset (downloaded from PopFly). This suggests DGRP strains with "uncertain" allele annotations at base Dmel.R5_2R_19488505 also bear the 2R_19488505_DEL indel. Bolstered by this finding, we scanned the *Drosophila* genome nexus for strings of uncertain variant calls (Ns) that affect at least 3 contiguous codons, treating these as putative indels. This may yield an undercount by ignoring smaller indels, or an overcount by treating regions bearing multiple SNPs in linkage as indels. Nevertheless, this annotation effort draws attention to strains bearing potential loss-of-function mutations in *Paillotin* that may be relevant to future studies. DPGP indel strain summaries are grouped by the location of the first disrupted codon (within the signal or mature peptide).

bioRxiv preprint doi: https://doi.org/10.1101/2025.05.12.653313; this version posted July 25, 2025. The copyright holder for this preprint (which was not certified by peer review) is the author/funder, who has granted bioRxiv a license to display the preprint in perpetuity. It is made available under aCC-BY 4.0 International license.

Mutation	Allele	Reference allele	Alternate allele	Effect	Present in
Pai$^{\Delta 41}$	*2R_19488505_DEL*	(C)CAAT CGGAG CGTCG TAGTA CAGTG GTCGT TGCTG TGAGC TTGGA GACCT GAACG GATTT CC	(C)-	61bp deletion	**DGRP:** 28, 31, 41, 176, 227, 321, 362, 390, 406, 409, 491, 707, 843, 852, 879, 892, 911<br>**DPGP:** CHB381
Pai$^{\Delta 370}$	*2R_19488690_SNP*	C	T	loss of start codon	**DGRP:** 370<br>**DPGP:** USW_59 possibly heterozygous
Pai^{G37*}	*Pai^{G37*}*	GGA codon	TGA codon	codon 37 premature stop	**DPGP:** KR7, KR4N
Pai$^{\Delta M1}$	*Pai$^{\Delta M1}$*	ATG codon	ANG codon	putative start codon disruption	**DPGP:** USI06
Signal peptide + DPase indel	*multiple*	ATGAA GCTGA TCGCA TTGTG CTGCC TGCTC CTTTT GGGCC TCCTG GGCTT CCTAG CTGCT CCCGG CGTCG CCTCG CCA	multiple	indel beginning within signal peptide	**DGRP:** 385, 887<br>**DPGP:** NTH_07, NTH_15, USW_26, USW_37, USW_62, USW_68, USW_49, USW_76, AUS_04, EA_70N, MW_6-2
Mature peptide indel not including Pai$^{\Delta 41}$	*multiple*	TCTCG CCACA CTGGA CCAGG AAACG GATCG GGATC TGGAG CTGGG TCCGG AAATC CGTTC AGGTC TCCAA GCTCA CAGCA ACGAC CACTG TACTA CGACG CTCCG ATTGG GAAAC CATCG AAGAC TATGT ACGCC TGA	multiple	indel beginning within mature peptide not including *Pai$^{\Delta 41}$*	**DPGP:** USW_74, USW_87, FR_2N

bioRxiv preprint doi: https://doi.org/10.1101/2025.05.12.653313; this version posted July 25, 2025. The copyright holder for this preprint (which was not certified by peer review) is the author/funder, who has granted bioRxiv a license to display the preprint in perpetuity. It is made available under aCC-BY 4.0 International license.

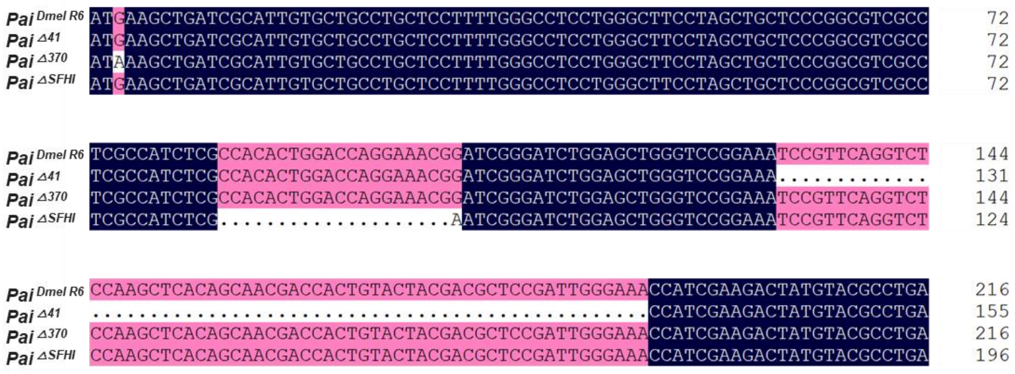

Figure S1. Nucleotide alignment of wild-type and mutant *Paillotin* alleles. Nucleotide sequences of wild-type *Paillotin* ($Pai^{Dmel\ R6}$) and the three mutant alleles ($Pai^{\triangle 41}$, $Pai^{\triangle 370}$, and $Pai^{\triangle SFHI}$) used in this study are aligned.

bioRxiv preprint doi: https://doi.org/10.1101/2025.05.12.653313; this version posted July 25, 2025. The copyright holder for this preprint (which was not certified by peer review) is the author/funder, who has granted bioRxiv a license to display the preprint in perpetuity. It is made available under aCC-BY 4.0 International license.

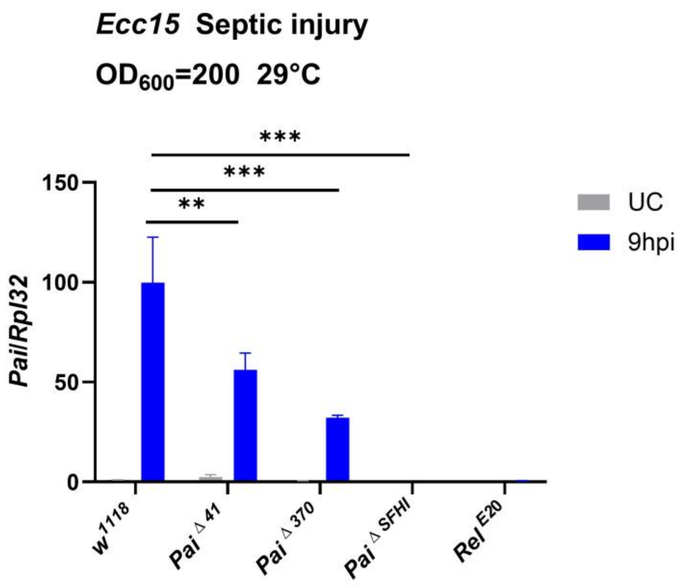

Figure S2: Expression of *Paillotin* in *Pai*$^{\Delta 41}$, *Pai*$^{\Delta 370}$, and *Pai*$^{\Delta SFHI}$ mutations. Expression was normalized to *w*^{1118} UC (unchallenged) flies set as a value of 1. The data shown in the figure are based on three independent experiments, with at least 30 flies per genotype at each time point in each experiment. Error bars represent SEM. Statistical analysis was performed using two-way ANOVA (** p<0.01, *** p<0.001).

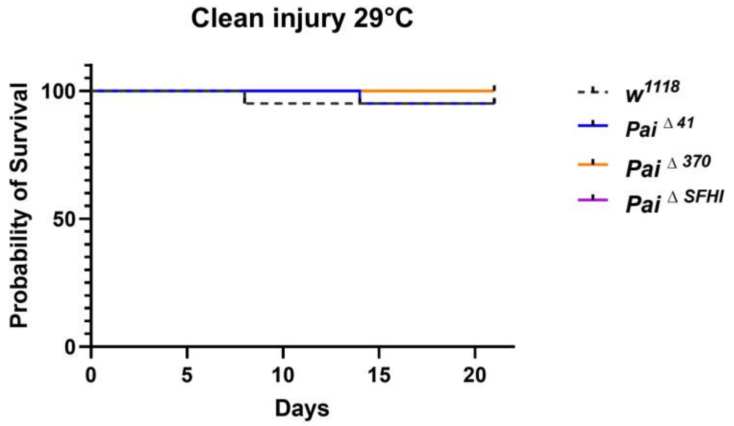

Figure S3: No susceptibility of *Paillotin* mutants to clean injury. The thorax of wild-type and mutants was pricked with a clean needle. A total of 80 flies were used in this experiment.

bioRxiv preprint doi: https://doi.org/10.1101/2025.05.12.653313; this version posted July 25, 2025. The copyright holder for this preprint (which was not certified by peer review) is the author/funder, who has granted bioRxiv a license to display the preprint in perpetuity. It is made available under aCC-BY 4.0 International license.

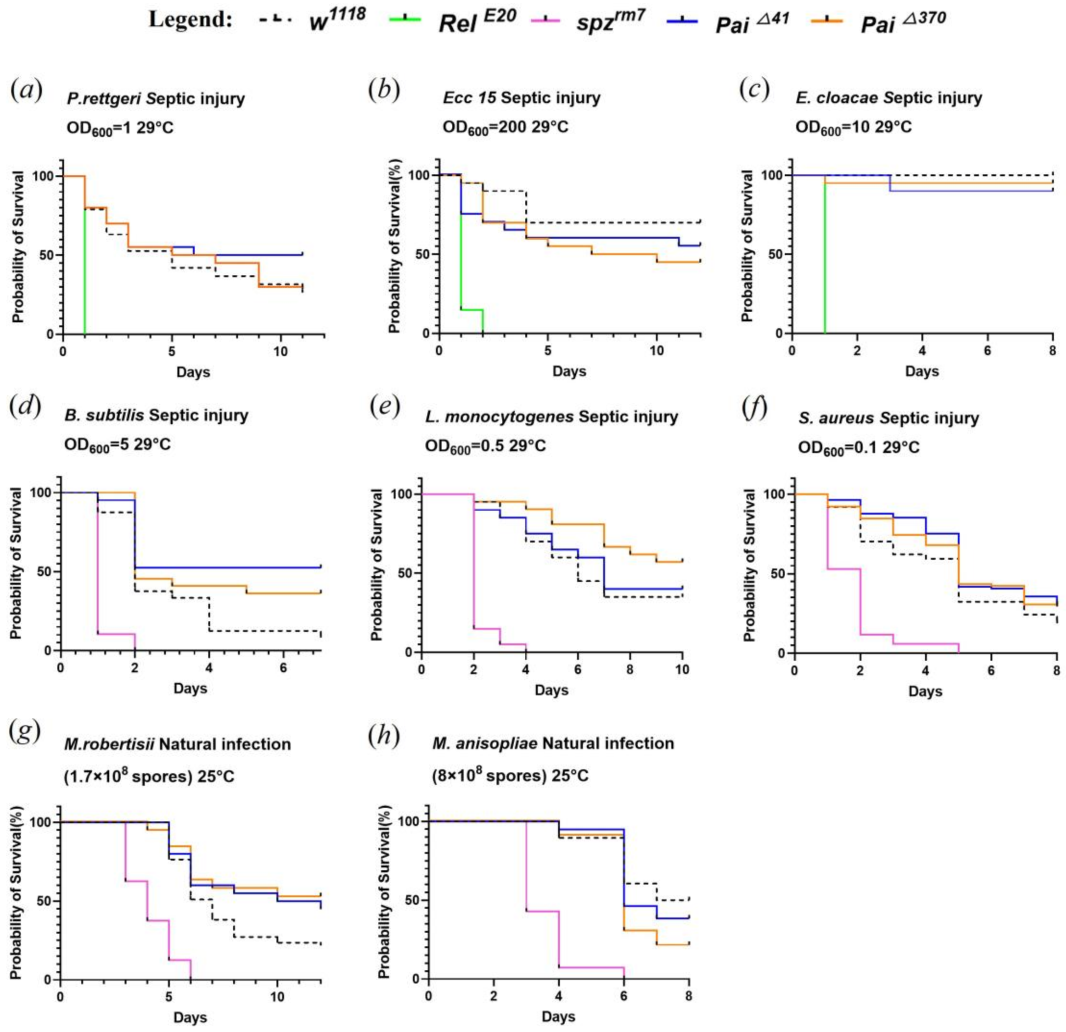

Figure S4: No consistent effect of *Paillotin* mutation on resistance to a broad spectrum of microbes. w^{1118} flies were utilized as wild-type controls, while spz^{rm7} flies lack the Toll pathway, and $Relish^{E20}$ flies lack the Imd pathway, were used as susceptible models for all survival experiments against infections. Tm and OD600 are indicated. Each survival assay was performed with a minimum of 80 flies.

bioRxiv preprint doi: https://doi.org/10.1101/2025.05.12.653313; this version posted July 25, 2025. The copyright holder for this preprint (which was not certified by peer review) is the author/funder, who has granted bioRxiv a license to display the preprint in perpetuity. It is made available under aCC-BY 4.0 International license.

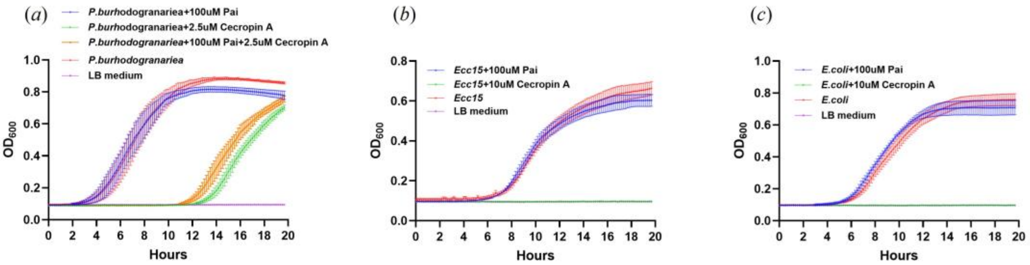

Figure S5: Paillotin did not show microbicidal activity *in vitro*. (*a*) The growth of *P. burhodogranariea* was monitored in the presence of the Paillotin peptide (100 µM) and/or the Cecropin A peptide (2.5 µM). The growth of *Ecc15* (*b*) and *E. coli* (*c*) was monitored in the presence of the Paillotin peptide (100 µM). Optical density (OD) values were measured every 10 minutes over 20 hours to generate bacterial growth curves. Different colored curves represent the conditions tested, as indicated in the figure legend.

bioRxiv preprint doi: https://doi.org/10.1101/2025.05.12.653313; this version posted July 25, 2025. The copyright holder for this preprint (which was not certified by peer review) is the author/funder, who has granted bioRxiv a license to display the preprint in perpetuity. It is made available under aCC-BY 4.0 International license.

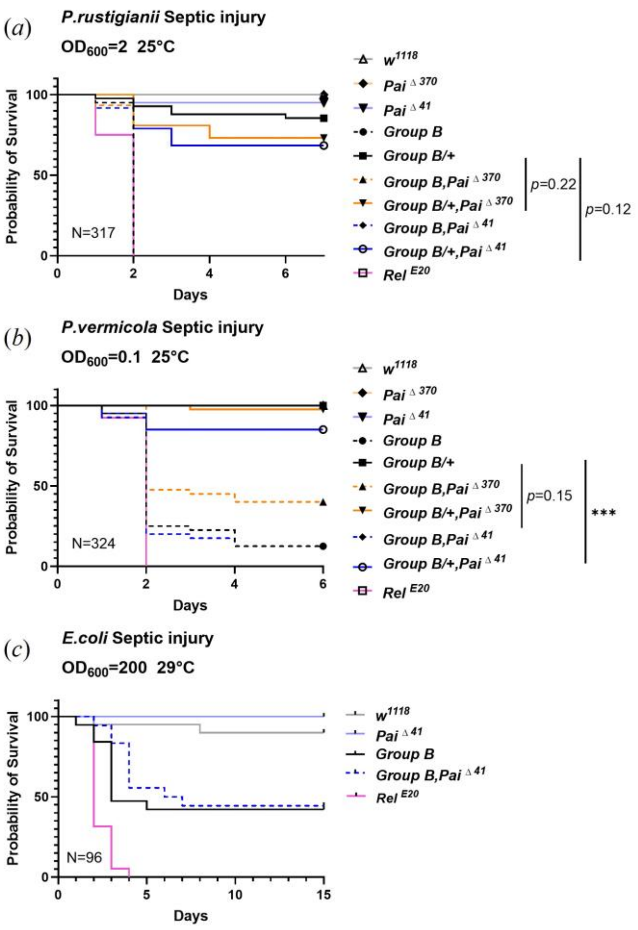

Figure S6: *Paillotin* shows a minor synergistic effect with *Group B* mutants against other *Providencia* species at the doses tested. (*a*) Both *Group B/+, Pai* compound mutants suffer a minor increase in mortality compared to *Group B/+* alone upon *Providencia rustigianii* infection. Although the synergistic effect between Paillotin and Group B was only marginally significant (Mutant, Cox Hazard Ratio (HR), *p*-value: *Paillotin*, HR =-0.01, p=0.904; *Group B*, HR =-2.8, p<0.001; *Paillotin***Group B*, HR = -0.41, p=0.09). (*b*) *Group B/+, Pai*$^{\Delta 41}$ mutants suffer a mortality increase compared to *Group B/+*. In addition, *Group B/+ Pai*$^{\Delta 370}$ shows no difference from *Group B/+*, but also *Group B, Pai*$^{\Delta 370}$ compound mutants survive better than *Group B* alone, indicating a complex phenotype after recombination for this infection that may mask the contribution of *Pai*$^{\Delta 370}$ at the chosen low-mortality dose (Mutant, Cox Hazard Ratio (HR), *p*-value: *Paillotin*, HR =0.15, p=0.14; *Group B*, HR =-2.7, p<0.001; *Paillotin***Group B*, HR = -1.13, p=0.03). Differences between *Pai*$^{\Delta 41}$ (truncated) and *Pai*$^{\Delta 370}$ (loss of start) may explain this difference. (*c*) No survival difference was observed for *Group B, Pai* compound mutants against *E. coli.* N=total number of flies in experiments.

bioRxiv preprint doi: https://doi.org/10.1101/2025.05.12.653313; this version posted July 25, 2025. The copyright holder for this preprint (which was not certified by peer review) is the author/funder, who has granted bioRxiv a license to display the preprint in perpetuity. It is made available under aCC-BY 4.0 International license.

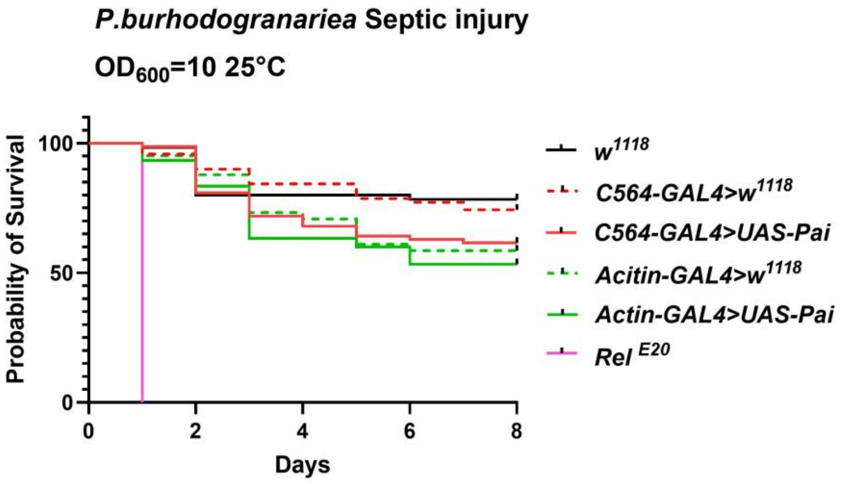

Figure S7: Overexpression of *Paillotin* does not confer protection against *P. burhodogranariea* infection in wild-type flies. Survival curves of flies overexpressing *Paillotin* by using *C564-Gal4* and *Actin-Gal4* drivers following septic infection with *P. burhodogranariea*. Survival assay was performed with a minimum of 120 flies.

bioRxiv preprint doi: https://doi.org/10.1101/2025.05.12.653313; this version posted July 25, 2025. The copyright holder for this preprint (which was not certified by peer review) is the author/funder, who has granted bioRxiv a license to display the preprint in perpetuity. It is made available under aCC-BY 4.0 International license.

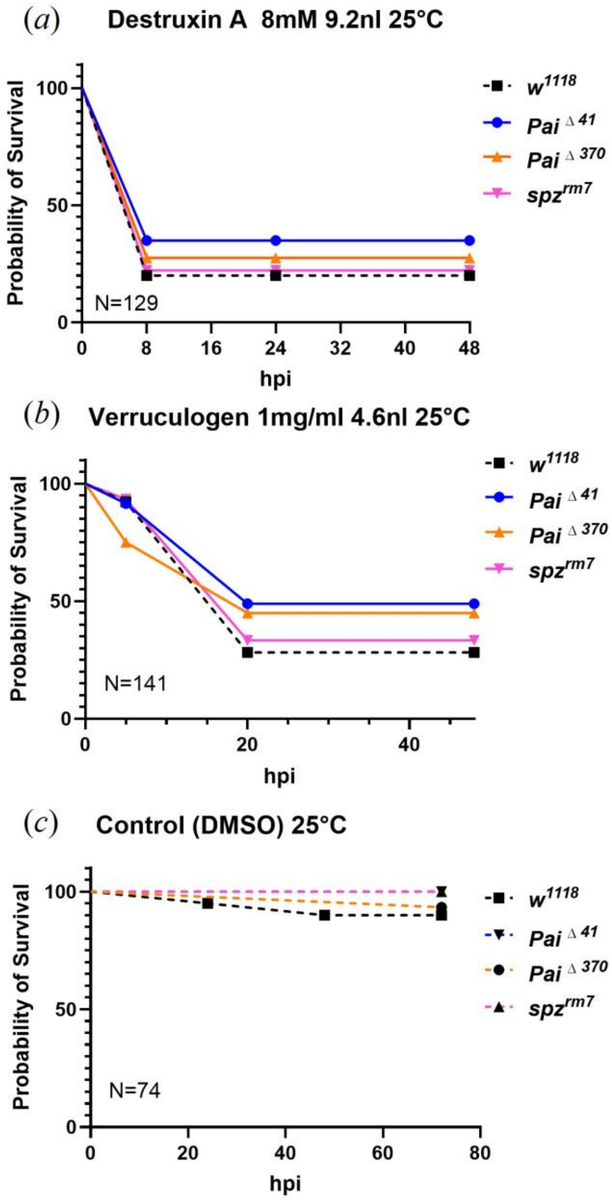

Figure S8: Paillotin does not contribute to protection against fungal toxins. (*a*) Survival of wild-type and *Paillotin* mutant flies following injection with 9.2 nl of 8 mM Destruxin A, a mycotoxin secreted by *M. robertsii*. (*b*) Survival of wild-type and *Paillotin* mutant flies injected with 4.6 nl of 1 mg/ml Verruculogen, a neurotoxin produced by *A. fumigatus*. (*c*) Survival of all tested fly strains following injection with DMSO as vehicle control. *spz*^{rm7} flies are used as positive controls. N=total number of flies in experiments.

bioRxiv preprint doi: https://doi.org/10.1101/2025.05.12.653313; this version posted July 25, 2025. The copyright holder for this preprint (which was not certified by peer review) is the author/funder, who has granted bioRxiv a license to display the preprint in perpetuity. It is made available under aCC-BY 4.0 International license.

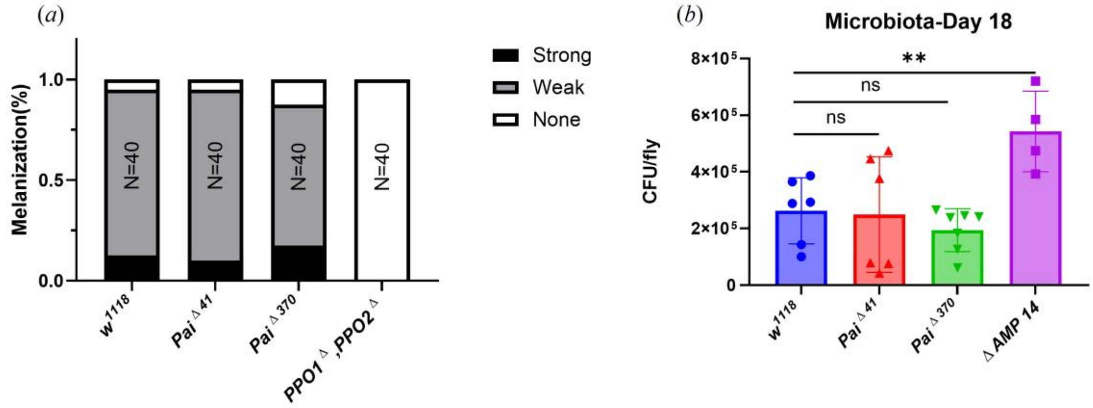

Figure S9: Paillotin is not involved in melanization response and microbiota regulation. (*a*) Cuticle blackening after clean injury is not impaired except in melanization-deficient flies (*PPO1*△*, PPO2*△). (*b*) CFUs per fly were measured for wild-type, *Paillotin* mutants, and the 14 AMP-deficient mutant (ΔAMP14). Data were analyzed using one-way ANOVA followed by Dunnett's multiple comparisons test with w^{1118} as the control. A significant increase in bacterial load was observed only in ΔAMP14, while no significant differences were detected between wild-type and *Paillotin* mutants. Data points represent the mean bacterial load per fly, derived from individual experiments using pools of five flies per genotype (*** $p<0.001$, n.s. = not significant, $p>0.05$).

bioRxiv preprint doi: https://doi.org/10.1101/2025.05.12.653313; this version posted July 25, 2025. The copyright holder for this preprint (which was not certified by peer review) is the author/funder, who has granted bioRxiv a license to display the preprint in perpetuity. It is made available under aCC-BY 4.0 International license.

Table S2: Drosophila strains used in this study.

Drosophila lines	Reference
iso; iso; Rel^{E20}	Hedengrenetal.,1999
iso; iso; spz^{rm7}	Lemaitre et al.,1996
iso; Bom$^{\Delta55}$*; iso*	Hanson et al., 2019
iso; iso; Rel^{E20}*, spz*^{rm7}	Ryckebusch et al., 2025
iso; Pai$^{\Delta41}$*; iso*	DGRP
iso; Pai$^{\Delta370}$*; iso*	DGRP
; Pai$^{\Delta SFHI}$*;*	Guangzhou Drosophila Resource Center
; ; UAS-Pai	This paper
iso ;Group B; iso	Hanson et al.,2019
iso ; PPO1$^{\Delta}$*,PPO2*$^{\Delta}$*; iso*	Bingelli et al., 2014
ΔAMP 14	Carboni et al., 2022
C564-Gal4	BDSC
Actin-Gal4	BDSC

bioRxiv preprint doi: https://doi.org/10.1101/2025.05.12.653313; this version posted July 25, 2025. The copyright holder for this preprint (which was not certified by peer review) is the author/funder, who has granted bioRxiv a license to display the preprint in perpetuity. It is made available under aCC-BY 4.0 International license.

Table S3: Microbial strains used in this study.

Type	Species	Strain,Source, DOI of previous study
Gram-negative	*Escherichia coli*	1106
	Pectobacterium carotovorum	Ecc15
	Providencia rettgeri	Dmel
	Enterobacter cloacae	B12
	Providencia burhodogranariea	B
	Providencia rustigianii	DSM 4541
	Providencia vermicola	DSM 17385
Gram-negative	*Micrococcus luteus*	doi:10.7554/eLife.44341
	Bacillus subtilis	168
	Enterococcus faecalis	254
	Listeria monocytogenes	BUG 1600
	Staphylococcus aureus	doi:10.7554/eLife.44341
Fungi	*Beauveria bassiana*	R444
	Metarhizium anisopliae	ARSEF-2575
	Metarhizium robertsii	ARSEF-2575
